# An Unexpected Case of Late Fatal Central Venous Catheter Sepsis: A Case Report

**DOI:** 10.1055/s-0040-1713415

**Published:** 2020-09-10

**Authors:** Rosanne Ottevanger, Sverre A.I. Loggers, Unsal Yapici, Joost M.R. Meijer, Giel G. Koning

**Affiliations:** 1 Department of Surgery, Noordwest Ziekenhuisgroep, the Netherlands; 2 Department of Pathology, Noordwest Ziekenhuisgroep, the Netherlands; 3 Department of Intensive Care, Noordwest Ziekenhuisgroep, the Netherlands; 4 Department of Surgery, Ikazia Hospital, Rotterdam, the Netherlands

**Keywords:** central venous catheter, sepsis, endovascular, infectious catheter, complication, vascular

## Abstract

**Introduction** Central venous catheters (CVC) are associated with risks and complications. Complications like vessel perforation, thrombosis, infection with significant morbidity and mortality, knotting, and ventricular perforation have been described. Another less-frequent complication is retained CVC fragments. We present a case of a very late but fatal complication after a CVC placement. This report is written in line with the consensus-based surgical case report guidelines (SCARE).

**Case** A 46-year-old male presented to the emergency department in a critical (septic) shock. The patients' medical history featured a long–intensive care admission 28 years ago. The cause of this sepsis was not evident until a computed tomography scan was performed to exclude a pulmonary embolism, revealing a remnant of a central catheter in both pulmonary arteries. Despite extensive resuscitation, the patient died within 24 hours after admission. An autopsy was performed confirming that the catheter remnant was the only possible cause of the fatal sepsis.

**Discussion** CVC's are associated with (fatal) complications; however, retainment of remnants are described unfrequently but do occur in almost 2% of the cases. Endovascular removal of these remnants has been performed successfully and should be the first treatment of choice if removal is considered. No evidence is available that suggests that routine removal has to be attempted but some longer term complications can be expected, so awareness of possible remnants after CVC removal should exist.

**Conclusion** Retained fragments of CVC's are rare but are described after prolonged use. This case shows that these retained intravascular fragments can cause fatal complications on the long-term. Upon removal of CVC's, there should be awareness that retainment of fragments can occur.

Sepsis is one of the leading causes of death worldwide. 1 2 Central venous catheter (CVC) associated blood stream infections are a major cause of morbidity and mortality in intensive care unit (ICU) patients and therefore one of the most feared complications of CVC placement. 3 4 Treatment is usually removal of the infected line and intravenous treatment with antibiotics. Retainment of CVC fragments after removal are rare and have been described after prolonged use. 5 Long-term fatal infectious complications of these CVC remnants have not been described yet. We present a rare case of a very late but fatal complication after a CVC placement according to the consensus based surgical case report guidelines (SCARE). 6

## Case Presentation

**Fig. 1:**
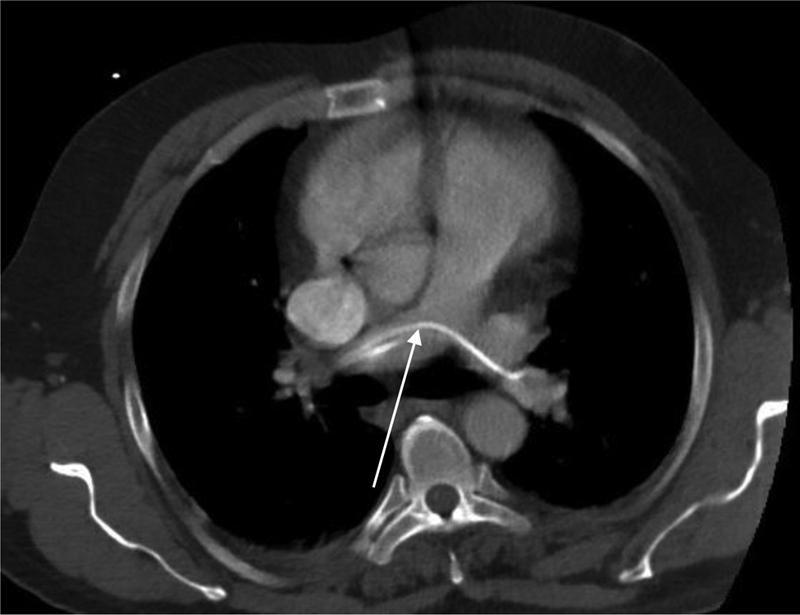
Transversal images of the CT-scan revealing the foreign body trapped in both pulmonal arteries, consistent with a CVC remnant. CT, computed tomography; CVC, Central venous catheters.

**Fig. 2:**
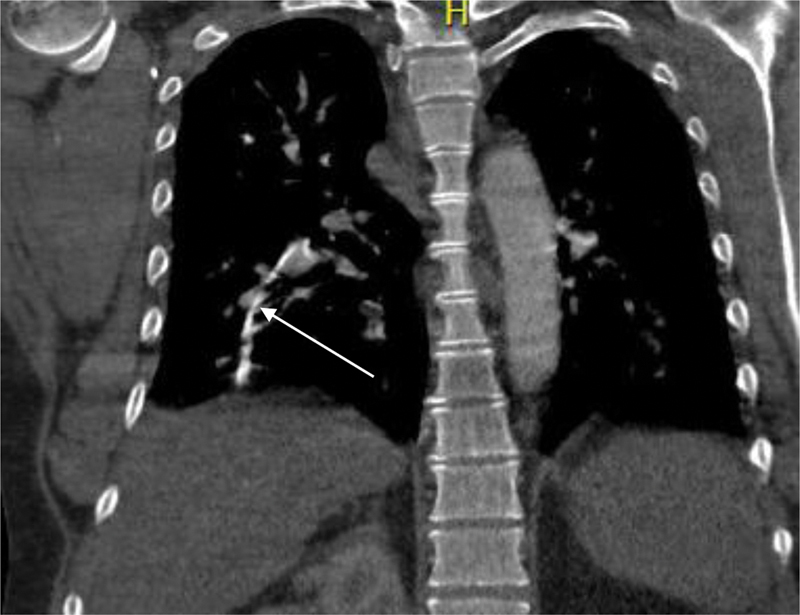
Coronal images of the CT-scan revealing the foreign body trapped in both pulmonal arteries, consistent with a CVC remnant with a possible false route in the right lung. CT, computed tomography; CVC, Central venous catheters.

We present a case of a 46-year-old male who presented to the emergency department (ED) in critical septic shock at our teaching hospital. Patient medical history featured type-II diabetes, alcohol abuse, pancreatitis, liver trauma requiring a laparotomy, and long-ICU admission in another hospital in 1989. Paramedics stated that patient was feverish for a couple of days and was found confused and sweating severely. No trauma had occurred. No further anamnesis was possible because of the current mental state. On physical examination, a very ill, pale, untended patient was seen with a tachycardia of 120 beats per minute, a blood pressure of 60/30 mm Hg, an 85% saturation and breathing frequency of 40 breaths per minute and a temperature of 36.0 °C. On physical examination, no external wounds or possible infection sites were found. The patient was treated with broad-spectrum intravenous antibiotics (ceftriaxone, metronidazole, and gentamicin), hydrocortisone, and fluids according to our sepsis treatment protocols. 7 The patient was marginally hemodynamically responsive and required norepinephrine to maintain an adequate mean arterial pressure. Blood results revealed a very severe metabolic acidosis, a severe acute kidney injury, and elevated inflammatory values were also found ( Table 1 ). A total body computed tomography (CT) scan was performed since the source of the sepsis remained unknown. The CT scan revealed a foreign body in both pulmonary arteries that was a possible remnant of an old central venous catheter with a length of approximately 20 to 25 cm on the CT ( Figs. 1 and 2 ) and an infrarenal dissection with no signs of organ hypoperfusion (probably of older origin). A quick-look echocardiogram showed a dilated right ventricle with good functions, unchanged since 2013. The patient was admitted to the ICU for further resuscitation and hemodynamic stabilization. Continuous venovenous hemofiltration was started via a dialysis catheter due to anuria. During the placement of this dialysis catheter in the left jugular vein, another foreign body was diagnosed by ultrasound that looked like possible remnants of the same intravenous catheter. The hypothesis was that the CVC remnant became dislodged during removal from the insertion site at the jugular vein and split in two different remnants over time and migrated from the vena cava superior through the right atrium and ventricle and settled in the pulmonal artery system.

**Table 1:** Laboratory values at admission and 18 hours after admission with maximum vasopressin, dobutamine, and norepinephrine administration

	Admission	After 18 hours	Reference values
pH	7.19	6.94	7.35–7.45
Bicarbonate	10 (10)	7 (7)	23–30 mmol/L (range: 21–28 mEq/L)
Lactate (arterial)	11.5 (104)	> 20 (180)	0.5–2.2 mmol/L (mg/dL)
C-reactive protein	142	152	0–5.0 mg/L
White blood cell count	2.3	11.1	4.0–10.0 × 10^9/L
Hemoglobin	8.2 (13.2)	8.0 (12.9)	8.5–11.0 mmol/L (range: 14.0–17.5 g/dL)
Creatinine	445 (5.84)	482 (6.32)	60–110 μmol/L (range: 0.6–1.2 mg/dL)
Urea	25.0 (70.0)	22.3 (62.5)	2.5–7.5 mmol/L (range: 8–23 mg/dL)
Glucose	2.1 (37.8)	8.2 (147.8)	3.3–7.8 mmol/L (range: 70–110 mg/dL)

**Fig. 3:**
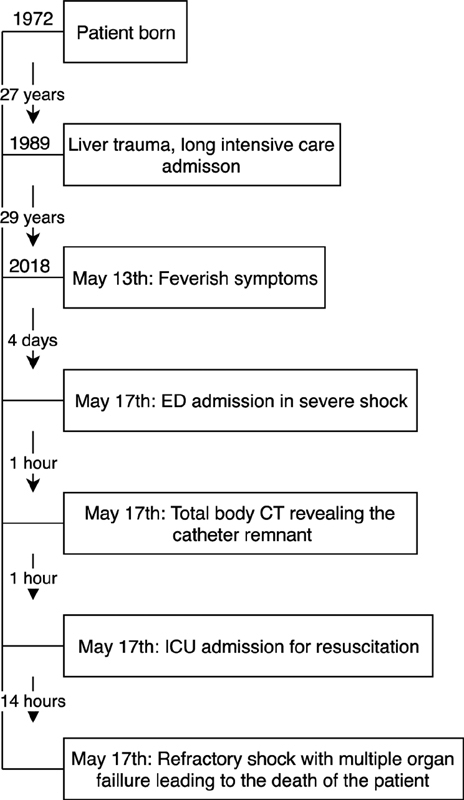
Timeline. CT, computed tomography; CVC, Central venous catheters; ED, emergency department; ICU, intensive care unit.

Despite the maximum use of extensive resuscitation and vasopressors, the patient hemodynamically and chemically worsened ( Table 1 ). The patient was to medically unstable to undergo further interventions in the form of surgery or endovascular retrieval of the catheter due to the hemodynamic instability. Within a few hours the patient developed end-stage multiorgan failure probably caused by the refractory sepsis with a possible component of obstructive shock due to the remnant, ultimately leading to death within 18 hours after admission ( Fig. 3 ). An autopsy was performed revealing the following: a remnant of a drain/catheter in both pulmonary arteries with a false route of 2 cm in the right lung with multiple adhesions to the arterial wall (most likely a 28-year-old CVC, dating back to the ICU admission in 1989). Microscopically, numerous neutrophils and bacterial colonies were seen and an *Enterococcus faecium* was cultured from the remnant and therefore the most probable cause of the sepsis. An enlarged heart with biventricular dilatation was also reported which could have contributed to the fast clinical deterioration because of preexistent heart failure (possibly alcoholic cardiomyopathy or due to more chronical obstruction in the pulmonal arteries by the remnant).

## Discussion

CVCs are associated with risks and complications 3 4 Complications like leakage, pneumothorax, occlusion, dislocation, vessel perforation, thrombosis, infection, and knotting are frequently described. 8 9 Less-frequent reported complications are retained CVC fragments as a result of mechanical complications, remnant migration, and cardiac perforations. 10 11 However, fatal sepsis as a cause of a migrated CVC remnant has, to our knowledge, not been described in the literature. Milbrandt et al describe 2% of line remnants after central venous catheter placement in 200 pediatric patients in their multicenter cohort study. With none of these patients developing any long-term (5.4 ± 3.9 years) symptoms, evidence of thrombus, infection, or catheter migration. 12 Bautista et al reported CVC remnants in 1.6% of the cases in their cohort of pediatric patients, with all of the CVC's used for a prolonged period of time in combination with chemotherapy. 5 The CVC remnants are probably caused by breakage of the lines because of fixation of the catheters by scar formation with calcification of the fibrin. 13 A systematic review by Surov et al reported on 215 cases of intravenous catheter embolization. The migration sites of the catheter fragments were the superior vena cava or peripheral veins (15.4%), the right atrium (27.6%), right ventricle (22%), and pulmonary arteries (35%). The main clinical signs of catheter embolization were catheter malfunction (56.3%), arrhythmia (13%), and pulmonary symptoms (4.7%). In almost 25% no symptoms occurred. No fatal septic complications did arise. 14

If there is a fracture of the line, remnant embolization has occurred or when these catheters are irretrievable because of fixation, the options are conservative treatment leaving the fragment in situ, attempt open surgical removal, or intravascular removal. Surgical removal would sometimes necessitate thoracotomy with venous reconstruction. 11 The endovascular approach has been proven to be feasible, safe, and effective for the management of mechanical complications of central venous devices in experienced hands. 10 15 Therefore endovascular retrieval should be considered the first approach when CVC remnants are detected to prevent aforementioned complications. However, in this case, the sepsis evaluated rapidly into death, endovascular options for removing a corpus alienum like this, which should be covered by fibrous tissue after all these years, seemed to be limited.

## Conclusion

Retained fragments after breakage of CVC's are rare but do occur. This case shows that these retained intravascular fragments can cause long-term and fatal complications. CVC systems should be handled with care and, upon removal, the integrity of the catheter's system should be checked. There should be awareness of retainment of fragments and the consequences with regards to possible (fatal) complications.
